# Metabolites of De Novo Purine Synthesis: Metabolic Regulators and Cytotoxic Compounds

**DOI:** 10.3390/metabo12121210

**Published:** 2022-12-02

**Authors:** Olga Souckova, Vaclava Skopova, Veronika Baresova, David Sedlak, Anthony J. Bleyer, Stanislav Kmoch, Marie Zikanova

**Affiliations:** 1 Department of Paediatrics and Inherited Metabolic Disorders, First Faculty of Medicine, Charles University and General University Hospital in Prague, Ke Karlovu 455/2, 128 08 Prague, Czech Republic; 2 CZ-OPENSCREEN: National Infrastructure for Chemical Biology, Institute of Molecular Genetics, Czech Academy of Sciences, 142 00 Prague, Czech Republic; 3 Section on Nephrology, Wake Forest School of Medicine, Winston-Salem, NC 27103, USA

**Keywords:** purine synthesis, PFAS, PAICS, ADSL, ATIC, cytotoxicity, FGAR, AIR, SAICAR, AICAR

## Abstract

Cytotoxicity of de novo purine synthesis (DNPS) metabolites is critical to the pathogenesis of three known and one putative autosomal recessive disorder affecting DNPS. These rare disorders are caused by biallelic mutations in the DNPS genes phosphoribosylformylglycineamidine synthase (PFAS), phosphoribosylaminoimidazolecarboxylase/phosphoribosylaminoimidazolesuccinocarboxamide synthase (PAICS), adenylosuccinate lyase (ADSL), and aminoimidazole carboxamide ribonucleotide transformylase/inosine monophosphate cyclohydrolase (ATIC) and are clinically characterized by developmental abnormalities, psychomotor retardation, and nonspecific neurological impairment. At a biochemical level, loss of function of specific mutated enzymes results in elevated levels of DNPS ribosides in body fluids. The main pathogenic effect is attributed to the accumulation of DNPS ribosides, which are postulated to be toxic to the organism. Therefore, we decided to characterize the uptake and flux of several DNPS metabolites in HeLa cells and the impact of DNPS metabolites to viability of cancer cell lines and primary skin fibroblasts. We treated cells with DNPS metabolites and followed their flux in purine synthesis and degradation. In this study, we show for the first time the transport of formylglycinamide ribotide (FGAR), aminoimidazole ribotide (AIR), succinylaminoimidazolecarboxamide ribotide (SAICAR), and aminoimidazolecarboxamide ribotide (AICAR) into cells and their flux in DNPS and the degradation pathway. We found diminished cell viability mostly in the presence of FGAR and AIR. Our results suggest that direct cellular toxicity of DNPS metabolites may not be the primary pathogenetic mechanism in these disorders.

## 1. Introduction

**Figure 1:**
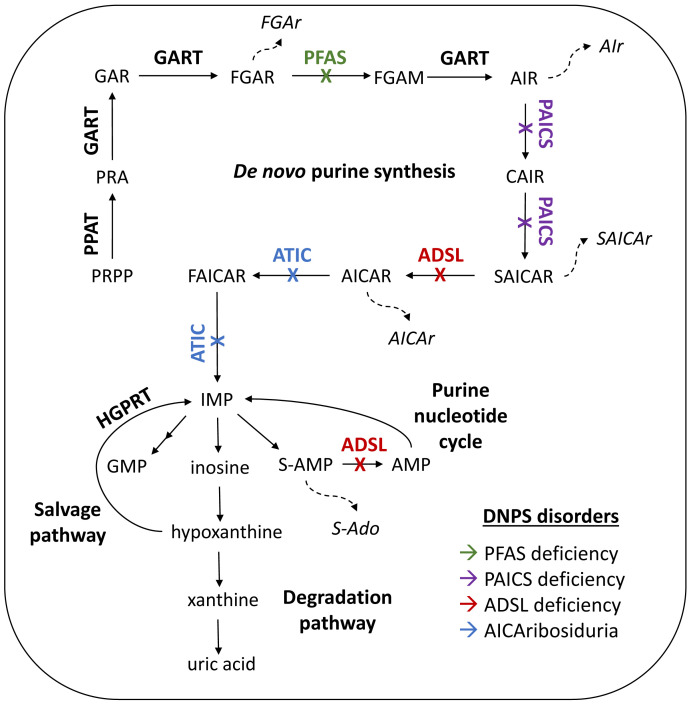
Purine metabolism. De novo purine synthesis (DNPS). Phosphoribosylpyrophosphate (PRPP) is processed by amidophosphoribosyl transferase (PPAT) to phosphoribosylamine (PRA). The 2nd, 3rd, and 5th steps are catalyzed by the trifunctional enzyme glycineamide ribonucleotide synthetase/aminoimidazole ribonucleotide synthetase/glycineamide ribonucleotide transformylase (GART). PRA results in glycineamide ribotide (GAR), from which formylglycineamide ribotide (FGAR) is created. The enzyme phosphoribosylformylglycineamidine synthase (PFAS) catalyzes transformation into formylglycineamidine ribotide (FGAM), which then forms aminoimidazole ribotide (AIR). AIR is processed by the bifunctional enzyme phosphoribosylaminoimidazolecarboxylase/phosphoribosylaminoimidazolesuccinocarboxamide synthase (PAICS) into carboxyaminoimidazole ribotide (CAIR) and succinylaminoimidazolecarboxyamide ribotide (SAICAR), respectively. The bifunctional enzyme adenylosuccinate lyase (ADSL) catalyzes the reaction of SAICAR to aminoimidazolecarboxamide ribotide (AICAR) in DNPS and succinyl-AMP (SAMP) to adenosine monophosphate (AMP) in the purine nucleotide cycle. The bifunctional enzyme aminoimidazole carboxamide ribonucleotide transformylase/inosine monophosphate cyclohydrolase (ATIC) catalyzes the last two steps that result in formamidoimidazolecarboxamide ribotide (FAICAR) and inosine monophosphate (IMP). IMP is precursor for synthesis of guanosine monophosphate (GMP) and AMP. Defects in DNPS enzymes lead to accumulation of ribosides, the dephosphorylated DNPS metabolites: FGAr, AIr, SAICAr, AICAr, succinyladenosine (SAdo). Enzymes connected with DNPS disorders are distinguished by colors: green for PFAS deficiency, purple for PAICS deficiency, red for ADSL deficiency, blue for AICAribosiduria. The main salvage enzyme of purines—hypoxanthineguaninephosphoribosyl transferase (HGPRT)—catalyzes conversion of hypoxanthine into IMP and guanine into GMP (not shown). Degradation of purines goes through IMP to inosine, hypoxanthine, xanthine to the final product uric acid.

There are three known and one putative autosomal recessive disorders of de novo purine synthesis (DNPS). These disorders are caused by biallelic mutations in the genes phosphoribosylformylglycineamidine synthase (PFAS), phosphoribosylaminoimidazolecarboxylase/phosphoribosylaminoimidazolesuccinocarboxamide synthase (PAICS), adenylosuccinate lyase (ADSL), and aminoimidazole carboxamide ribonucleotide transformylase/inosine monophosphate cyclohydrolase (ATIC), resulting in the disorders PFAS deficiency, PAICS deficiency, ADSL deficiency, and AICAribosiduria, respectively (Figure 1) [1,2,3,4,5].

PAICS deficiency, ADSL deficiency, and AICAribosiduria lead to developmental abnormalities, psychomotor retardation, and neurological impairment, and in severe cases, cause death [2,3,4,5]. While the pathophysiology of these conditions is uncertain, it is postulated that the accumulation of dephosphorylated metabolites (ribosides) of the corresponding enzymes in blood, urine, and cerebrospinal fluid (CSF) [5,6] leads to clinical manifestations [5,7,8,9]. In healthy individuals, there is no detectable or a very low concentration of ribosides in body fluids [6,8,10], while affected individuals with DNPS disorders have high concentrations of formylglycineamide riboside (FGAr), succinylaminoimidazolecarboxamide riboside (SAICAr), succinyladenosine (SAdo), and aminoimidazolecarboxamide riboside (AICAr) in body fluids. Patients with ADSL deficiency have elevated urinary SAICAr (188 mmol/mol creatinine (median, n = 14), normal range: undetectable) and SAdo (259 mmol/mol creatinine (median, n = 14), normal range: 0.6–7.4 mmol/mol creatinine (n = 104)) and elevated CSF concentrations of SAICAr (212 µM) and SAdo (278 µM) (median, n = 28) [11]. Patients with AICAribosiduria (deficiency of ATIC) have slightly elevated urinary concentrations of SAICAr (59 mmol/mol creatinine (median, n = 2)) and SAdo (45 mmol/mol creatinine (median, n = 3)), and elevated urinary AICAr (212 mmol/mol creatinine (median, n = 4)) [2,4,11]. While little is known about the intracellular concentration of DNPS metabolites, DNPS enzyme activities are reduced in the tissues of affected patients [2,3,8,12,13,14]. In addition, since the concentration of nucleotide pools of adenine and guanine in patients remains unchanged, it has been hypothesized that the accumulation of DNPS ribosides causes cellular toxicity and is central to the pathogenesis of DNPS disorders [6,8,14,15]. Under normal conditions, DNPS enzymes assemble into a multienzyme complex called a purinosome, which facilitates the rapid channeling of metabolites [16]. When the purinosome cannot be assembled properly due to genetic defects in DNPS enzymes, the ability to carry out reactions and remove undesired toxic metabolites is impaired [16,17,18,19,20,21,22]. In addition, it has been speculated that succinylpurines bind to adenosine receptors. Despite its similarity to adenosine, no interaction of succinylpurines with receptors was found in the rat cerebral cortex fraction [23,24], and no toxic effects were found in rat cortical neurons [12]. Above that, DNPS metabolites may have unique physiologic functions. For instance, AICAr has been found to block the cell cycle, AICAribotide (AICAR) activates AMPK kinase, and SAICAribotide (SAICAR) stimulates pyruvate kinase isoform M2 (PKM2) in cancer cells [25,26,27,28,29,30,31,32]. Abnormal activation of DNPS in cancer cells has been demonstrated in hepatocellular carcinoma, lung adenocarcinoma, bladder tumorigenesis, and breast and prostate cancers where high expression of DNPS enzymes has been found. Downregulating the genes coding these particular enzymes results in decreased nucleotide pools, cell viability, proliferation, cell cycle arrest, and increased apoptosis. Although many interaction partners have been studied, the influence of DNPS metabolites remain unclear [33,34,35,36,37,38,39,40,41]. Therefore, the high flux of DNPS metabolites in cancer cells makes them a suitable model for studying the effect of purine intermediates that accumulate when this pathway is disrupted [42].

Because the pathogenetic effects of metabolites resulting from DNPS disorders have not been fully characterized, we examined cellular uptake and flux of the isotopically labeled metabolites ^13^C_2_—formylglycineamide ribotide (FGAR*), ^13^C_2_,^15^N—aminoimidazole ribotide (AIR*), ^13^C_2_,^15^N_2_—SAICAR (SAICAR*), and ^13^C_2_,^15^N_2_—AICAR (AICAR*). We also tested the cytotoxicity of the phosphorylated metabolites (ribotides) and ribosides FGAR/FGAr, AIR/AIr, SAICAR/SAICAr, and AICAR (or ZMP)/AICAr in different cancer and primary cell lines and determined their half-maximum inhibitory concentrations (IC_50_).

Based on our finding, we propose a model explaining the probable cytotoxic effect of DNPS metabolites. As DNPS enzymes are ubiquitously expressed in all tissues, the main pathogenic effect of DNPS disorders can be attributed to disruption of purinosome assembly [18,42] and the cytotoxic effects of the accumulation of ribosides that are excreted from cells by nucleoside transporters to the extracellular environment [43,44]. Exported ribosides can affect other cells through interaction with receptors and activation of a cascade of reactions within the cell, causing deviations from normal cellular homeostasis.

## 2. Materials and Methods

### 2.1. Cell Lines

HeLa cells were obtained from the Department of Pediatrics and Inherited Metabolic Disorders (DPIMD). Control skin fibroblasts were derived from primary tissue obtained from a healthy individual under informed consent and were the property of DPIMD. HepG2 and K562 were kindly provided by OpenScreen, IMG, Czech Republic. CAD-2A2D5 (CAD 5) cells derived from Cath a-differentiated (CAD) cells were provided by Sukhvir Mahal (The Scripps Research Institute, FL, USA). We previously prepared HeLa GART knock-out (KO), HeLa ADSL KO, HeLa ATIC KO (18) and similarly prepared HeLa GART-ADSL KO cells (GART: c.367–368insA/c.368delA; ADSL: c.105–131del/c.105–131del). We maintained primary skin fibroblasts, HeLa, HepG2, and K562 cells in Dulbecco’s Minimum Essential Medium supplemented with 10% FBS (Gibco, ThermoFisher Scientific, Waltham, MA, USA), 1% penicillin/streptomycin (Sigma-Aldrich, Merck, Darmstadt, DE). We maintained HeLa KO cells in DMEM supplemented with 10% FBS, 1% penicillin/streptomycin, 30 μM adenine [42]. We maintained CAD5 cells in modified Eagle’s Minimum Essential Medium (Opti-MEM/Reduced Serum Media, Gibco, ThermoFisher Scientific) supplemented with 10% FBS, 1% penicillin/streptomycin without phenol red. Cells were grown at 37 °C using a 5% CO_2_ incubator.

### 2.2. Chemicals

We purchased AICAR and adenine (Sigma-Aldrich, Merck). The chemicals: FGAR, FGAr, AIR, AIr, SAICAR, SAICAr, AICAr were prepared in our laboratory [42,45]. FGAR was purified on a weak anion-exchange polymeric sorbent Strata X-AW (Phenomenex, Torrance, CA, USA) in potassium phosphate buffer, pH 3.2 and eluted by methanol. AIR was purified on weak anion-exchange sorbent Strata X-AW (Phenomenex) at pH 5.3. SAICAR was purified on Strata X-AW at pH 5.1 or at Dowex^®^ 50W (Sigma-Aldrich, CZ) [45]. All ribosides were prepared from purified ribotides in reaction with calf intestine alkaline phosphatase (CIP) (NEB, Ipswich, MA, USA) as published previously [42]. CIP was eliminated from reaction mix by centrifugation through Amicon^®^ 3K column (Sigma-Aldrich, Merck). ^13^C_2_—FGAR (FGAR*); ^13^C_2_—FGAr (FGAr*); ^13^C_2_,^15^N—AIR (AIR*); ^13^C_2_,^15^N_2_—SAICAR (SAICAR*) were prepared and purified analogously with starting metabolites ^13^C_2_—glycine (Isotec, Merck) and ^13^C_2_,^15^N—glycine, ^15^N—glutamine, ^15^N—aspartate (Sigma-Aldrich, Merck). ^13^C_2_,^15^N_2_—AICAR (AICAR*) was prepared enzymatically from SAICAR* and purified on Strata X-AW at pH 5.1 followed by Dowex^®^ 50W in 0.1 M HCl and finally eluted with ammonium hydroxide. Metabolites absorbing UV light were analyzed via HPLC-DAD and the purity was set up to 95%. FGAR and FGAr do not absorb UV light and were analyzed via HPLC-DAD and LC-MS/MS, where only trace amounts of N^10^-formyl-THF, glycineamide ribotide (GAR), and GAr were detected.

### 2.3. Flux Experiment with Isotopically Labeled Metabolites and LC-MS/MS Measurement

We grew HeLa control and ATIC KO cells in a 6-well plate in purine-depleted medium overnight to stimulate DNPS. For the experiment, we fed cells with 24 μM FGAR*, 24 μM FGAr*, 25 μM AIR*, 30 μM SAICAR* or 25 μM AICAR* and ATIC KO cells with 35 μM AIR*, 30 μM SAICAR*, 25 μM AICAR* prediluted in purine-depleted medium. We incubated cells with SAICAR* and AICAR* for 3 h; AIR* for 6 h in HeLa control cells and for 3 h in ATIC KO cells, respectively; FGAR* for 1 h, 3 h, 6 h, 24 h in HeLa control cells; FGAr* for 6 h in HeLa control and ATIC KO cells. We incubated cells with corresponding metabolite in purine-depleted medium at 37 °C using a 5% CO_2_ incubator. A total of 1 mL of medium was collected in a 1.5 mL tube and the cells were harvested by trypsinization. Cell lysate was prepared as previously described [42]. We prepared the samples for analysis by adding 150 μL of 80% ice cold methanol to 50 μL of cell lysate and 300 μL of ice cold 80% methanol to 100 μL of medium and let them incubate overnight at −80 °C. The samples were centrifuged, and the supernatant was dried using a speed-vac system and pellets were resuspended in buffer A [42] to the original volume. We analyzed samples for products of DNPS, salvage, and degradation pathway by LC-MS/MS as previously described with the modifications of transition states [42,46] (Table S1) and adjusting the gradient to shorter column: Prontosil 120-3-C18 AQ (150 × 3.0, 3 μm) (Bischoff chromatography, Leonberg, DE). Briefly, gradient elution was initiated with 2.5 min of 100% A (0.1% formic acid), followed by a linear increase to 20% of B (0.1% formic acid in acetonitrile) for 6.5 min, then an increase to 60% of B for next 1 min and followed by regeneration of the column. The flow rate was 0.3 mL/min for the first step and then increased to 0.4 mL/min. The limit of detection (LoD) was defined using a signal-to-noise ratio of 3:1. The peak area corresponding to the naturally occurring isotope of the metabolite was calculated from the peak area of the unlabeled metabolite [47] and subtracted from the area of the labeled metabolite.

Representative chromatograms of the labeled metabolites detected in cells and medium treated with AIR* are shown in Figure S3.

### 2.4. Cell Viability in the Presence of DNPS Metabolites: FGAR, FGAr, AIR, AIr, SAICAR, SAICAr, AICAR, AICAr in HeLa, CAD 5, HepG2, K562, Skin Fibroblasts, HeLa GART KO, ADSL KO, GART-ADSL KO Cells

Experiments with HeLa, HepG2, K562 and skin fibroblast cells were performed in normal DMEM without phenol red or Opti-MEM without phenol red (for CAD 5 cells) and supplemented with 10% FBS, 2 mM Glutamax, 1 mM pyruvate, and 1% penicillin/streptomycin (Gibco, ThermoFisher Scientific). We seeded cells (7 × 10^2^) in 24 μL total volume per well in a 384-well plate format. Cells grew in 12 μL of medium overnight at 37 °C using a 5% CO_2_ incubator. The following day, we prediluted individual metabolites: FGAR, FGAr, AIR, AIr, SAICAR, SAICAr, AICAR, AICAr, in a medium in a series of 14 to reach final concentrations between 1.7 μmol/l and 1 mmol/L. We added 12 μL of prediluted metabolite to the cells to achieve the final concentration in a total volume of 24 μL per well. We assessed the luminescence signal after 72 h of cultivation by determining the level of intracellular ATP with the ATPliteTM (PerkinElmer, Waltham, MA, USA) luminescence system (PerkinElmer). The luciferase-catalyzed reaction of ATP and luciferin resulted in light production, and the ATP concentration (representing cell viability) was proportional to emitted light. Higher intracellular ATP levels correspond to metabolically active—and therefore viable—cells, while its decreased levels indicate attenuation of cellular metabolism, leading eventually to cell death accompanied by ATP depletion. Cytotoxicity is defined as the property of compounds that have deleterious effects on cells, thereby opposing their viability. we compared the detected activity in the viability assay with controls including untreated cells or a growth medium without cells. This allowed us to assess whether the observed viability corresponds to the level of healthy cells or whether it is reduced to a level where the sample does not contain live cells. We measured cell viability after addition of succinyl-AMP (SAMP) (not shown in paper), a substrate that is directly converted into AMP in ADSL reaction. We did not detect any changes in viability of HeLa control cells, which also indicates that the selected method is robust and no ‘artificial’ synthesis of ATP occurs.

We added 11 μL of ATPlite solution to a total volume of 24 μL of medium with cells and shook plates on an orbital shaker for 15 min at room temperature. We recorded the luminescence signal on an EnVision plate reader (PerkinElmer). We analyzed data and calculated IC_50_ values for each sample using Microsoft Office Excel and the GraphPad Prism software.

Experiments with GART KO cells were performed in purine-depleted DMEM without phenol red, supplemented with 10% dialyzed FBS, 2 mM Glutamax, 1 mM pyruvate, and 1% penicillin/streptomycin. The individual metabolites were prediluted in dialyzed FBS: FGAR, AIR, SAICAR, AICAR, IMP at the final six concentrations ranging from 5 μmol/L to 0.5 mmol/L. We added 12 μL of prediluted metabolite to HeLa GART KO and measured cell viability after 1 day, 3 days, and 5 days of cultivation.

Experiments with HeLa control, GART KO, ADSL KO, GART-ADSL KO cells were performed in normal DMEM without phenol red and supplemented with 10% FBS, 2 mM Glutamax, 1 mM pyruvate, and 1% penicillin/streptomycin, and with 0 or 200 μM AICAR. Cell viability was measured after 72 h of cultivation.

## 3. Results

### 3.1. Distribution of DNPS Metabolites within Cells

For analysis of uptake and flux of DNPS metabolites, specifically FGAR, AIR, SAICAR, and AICAR, we used HeLa control cells that have fully functional DNPS, and HeLa ATIC KO cells that have impaired DNPS in the last ATIC enzyme and accumulate AICAR—and to a lesser extent, other metabolites—upstream of the impaired ATIC enzyme [42].

**Figure 2:**
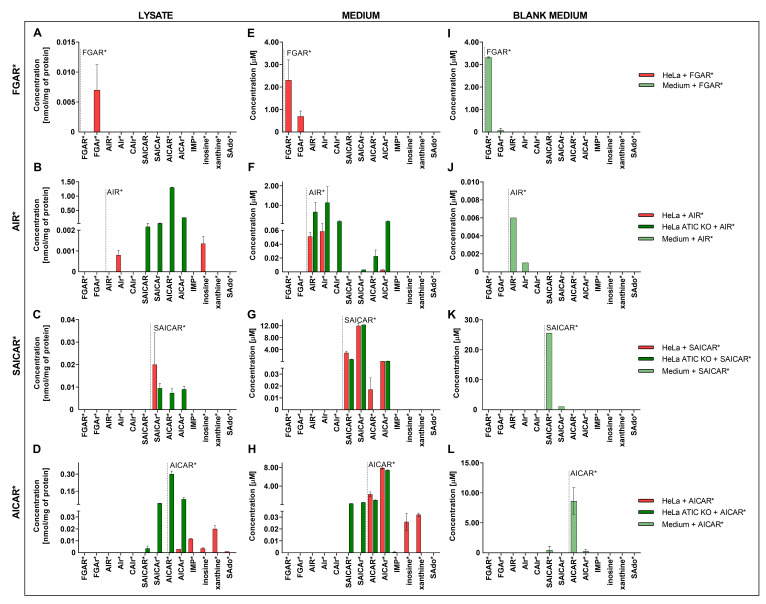
Accumulation of isotopically labeled DNPS metabolites (FGAR*, AIR*, SAICAR*, AICAR*). Vertical dashed line indicates which DNPS metabolite was added. The graphs (**A**–**D**) represent the concentration of detected isotopically labeled metabolites in HeLa control cell lysate after the addition of FGAR*, AIR*, SAICAR*, and AICAR* (red), and in ATIC KO cell lysate after addition of FGAR*, AIR*, SAICAR*, and AICAR*(dark green). The graphs (**E**–**H**) represent the concentration of detected isotopically labeled metabolites in HeLa control cell growth medium after the addition of FGAR*, AIR*, SAICAR*, and AICAR* (red), and in ATIC KO cell growth medium after addition of FGAR*, AIR*, SAICAR*, and AICAR* (dark green). The graphs (**I**–**L**) display results for detected labeled metabolites in blank medium (medium without cells) after addition of FGAR*, AIR*, SAICAR*, and AICAR* (light green). The results for untreated cells were zero or approaching zero, and are not shown in the graph. Each data point represents the mean of a single experiment measured in duplicate. Vertical bars represent S.D. (n = 2). List of all measured isotopically labeled metabolites is shown in Table S1.

We exposed HeLa control and HeLa ATIC KO cells to 24–35 μM of the isotopically labeled DNPS metabolites ^13^C_2_-FGAR (FGAR*); ^13^C_2_-FGAr (FGAr*); ^13^C_2_, ^15^N-AIR (AIR*); ^13^C_2_, ^15^N_2_-SAICAR (SAICAR*); ^13^C_2_, ^15^N_2_—AICAR (AICAR*) (Figure 2A–L; Figure S1A–G) and measured resulting metabolites by the LC-MS/MS method in cell lysate and growth media.

After treatment of HeLa control cells with FGAR*, we detected only FGAr* in cell lysate and growth medium (Figure 2A,E,I, Figures S1A and S2A). As we did not detect any FGAR* or downstream DNPS product in the cell lysate in different time points, we hypothesize that FGAR* did not enter the DNPS and that it dephosphorylates at the cellular membrane. Further, we treated HeLa control cells and ATIC KO cells with FGAr* to see whether it is phosphorylated and processed inside the cell. We detected only FGAr* in growth medium and cell lysate, which corresponds with the hypothesis (Figure S2B).

We monitored the processing of AIR* and detected AIr* and inosine* in HeLa control cell lysate and AIr*, and AICAr* in growth medium (Figure 2B,F,J and Figure S1B). Next, we performed the same experiment with ATIC KO cells. As we expected, SAICAR*, AICAR*, and their ribosides appeared in cell lysate and AIr*, CAIr*, SAICAr*, and AICAr* appeared in growth medium (Figure 2B,F,J and Figure S1C). Surprisingly, we detected AICAR* in growth medium of ATIC KO cells. Because we did not see AICAR* in growth medium of HeLa control cells or in blank medium, we believe that the massive accumulation of AICAR in ATIC KO cells facilitates its transport into media, suggesting the existence of transporters for DNPS metabolites. The results indicate that AIR entered the purinosome and was metabolized by DNPS.

SAICAR* administered to HeLa control cell medium resulted in the production of SAICAr* in HeLa cell lysate and SAICAr*, AICAR*, and AICAr* in growth medium (Figure 2C,G,K and Figure S1D). When we treated ATIC KO cells with SAICAR*, we were able to measure SAICAr*, AICAR*, and AICAr* in the cell lysate and SAICAr* and AICAr* in growth medium (Figure 2C,G,K and Figure S1E). As we detected only AICAR* and AICAr* in HeLa control cells and no other downstream products, we hypothesize that SAICAR* may not be a constituent of the purinosome, but only the free ADSL enzyme.

AICAR* added to HeLa control cell medium resulted in identification of the DNPS metabolites AICAr* and IMP*; metabolites from the salvage and degradation pathway, inosine* and xanthine*; and dephosphorylated metabolite from the purine nucleotide cycle (PNC)–SAdo*, in the cell lysate (Figure 2D and Figure S1F). In growth media, we identified the metabolites AICAr*, IMP*, inosine*, and xanthine* (Figure 2H,L and Figure S1F). We carried out the experiment in ATIC KO cells and detected AICAR*, AICAr*, and surprisingly, SAICAR* and SAICAr* in cell lysate and AICAr* and SAICAr* in the medium (Figure 2D,H,L and Figure S1G). Based on these results, we hypothesize that AICAR* metabolizes through DNPS in HeLa control cells. Moreover, the ATIC KO cells have a strong reverse reaction of the ADSL enzyme that turns AICAR* into SAICAR*.

### 3.2. Cell Viability in the Presence of DNPS Metabolites

Having established the fate of DNPS metabolites in the cells, we proceeded to study the effect of DNPS metabolites on cell viability. The goal was to determine whether DNPS metabolites affect cell viability and whether there is a difference in the impact between phosphorylated and dephosphorylated forms. To test the effects of the studied DNPS metabolites on cellular viability, we used five different cell lines: human cervix epithelioid carcinoma cells (HeLa), Cath-a differentiated catecholaminergic cells (CAD 5), hepatocyte carcinoma cells (HepG2), chronic myelogenous leukemia cells (K562), and primary skin fibroblasts. We established the IC_50_ values in each cell line if applicable and found that the metabolites of the first part of DNPS–FGAR and AIR had stronger inhibitory effects on cell viability than SAICAR and AICAR. We also found that ribosides had more severe impact on cell viability than their phosphorylated analogues (Table 1). Nevertheless, none of these metabolites showed strong cytotoxicity, as the general threshold for cytotoxicity is less than 100 μM concentration.

**Figure 3:**
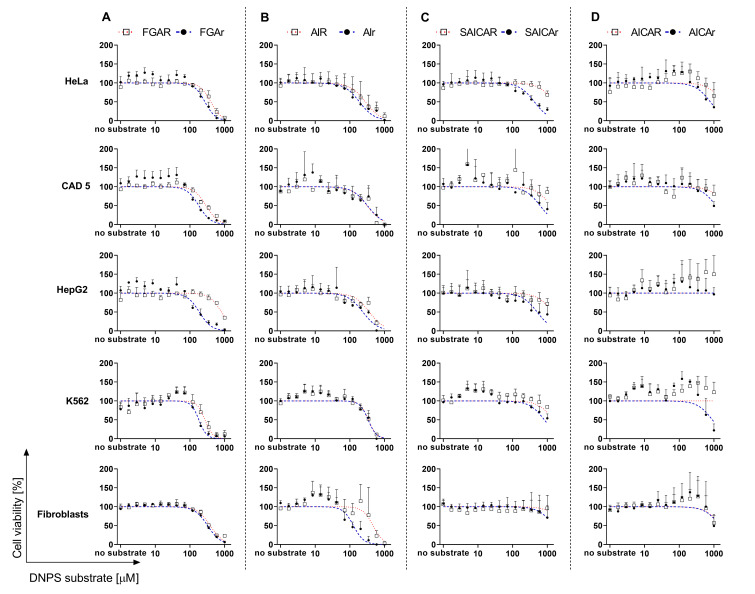
Cytotoxicity of DNPS metabolites (FGAR/r, AIR/r, SAICAR/r, AICAR/r) in HeLa, CAD 5, HepG2, K562 cells and skin fibroblasts. Cells were cultured in normal growth medium containing corresponding phosphorylated (squares, red dotted line) or dephosphorylated (black circles, blue dashed line) DNPS metabolites at 14 concentrations ranging from 1.7 μmol/l to 1 mmol/L, and cell viability was evaluated 72 h later. Viability curves of (**A**) FGAR and FGAr, (**B**) AIR and AIr, (**C**) SAICAR and SAICAr, and (**D**) AICAR and AICAr were established. The *X*-axis displays the concentration of the DNPS metabolite [μM] and the *Y*-axis displays the cell viability [%]. The viability level of untreated cells was set as reference and data were normalized to untreated cells and IC_50_ values (Table 1) were calculated using GraphPad Prism software. Each data point represents the mean of 3 independent experiments. Vertical bars represent S.D. (n = 3).

We estimated IC_50_ values for FGAR and FGAr (Table 1). According to our findings, FGAR and FGAr have a toxic effect for most tested cell lines (Figure 3A).

However, HepG2 cells showed low susceptibility to FGAR treatment. The response of K562 cells to treatment of FGAR and FGAr showed both increased (FGAR and FGAr 24–70 μmol/L) and decreased viability (270 and 181 μmol/L, respectively). When a low dose of a drug enhances the cell viability while a high dose of a drug diminishes cell viability, it is called hormetic dose–response (biphasic effect). This phenomenon is commonly observed in drug development as well as in the behavior of some cancer cell lines [48].

We previously published the strong effect of AIr on the viability of CAD 5 cells and skin fibroblasts [3]. In the presented study, we tested three additional cell lines—HeLa, HepG2, and K562—and we observed decreased viability of each cell line after treatment with AIR or AIr (Figure 3B).

Interestingly, the effect of SAICAR on cell viability was not significant, and we detected low cell viability only at concentrations of 1 mmol/L in most cell lines (Figure 3C). The impact of SAICAr on cell metabolism varied and exhibited the most severe impact in the HeLa cell line. This finding was supported by previously published results of SAICAr treatment in CAD 5 cells and skin fibroblasts where both inhibitory and stimulatory effects were observed [3]. We detected an increased viability in K562 cells treated with SAICAR and SAICAr at concentrations below 24.4 μmol/L. On the other hand, HepG2 and skin fibroblasts did not show a strong response to treatment (Figure 3C).

AICAR and AICAr lowered cell viability only at >600 µmol/L concentration for most cell lines (Figure 3D). Due to stimulatory response, the IC_50_ values were not estimated or estimated to be greater than 1.6 mmol/L for AICAR and 0.8 mmol/L for AICAr (Table 1). We were able to observe the biphasic effect of AICAr on the growth of CAD 5 and K562 cells. Furthermore, AICAR supported the growth of HepG2 and K562 cells at a concentration below 1 mmol/L, and we found similar behavior in HeLa cells and skin fibroblasts at concentrations greater than 346 μmol/L.

### 3.3. AICAR Restores the Viability of HeLa GART KO Cells

**Figure 4:**
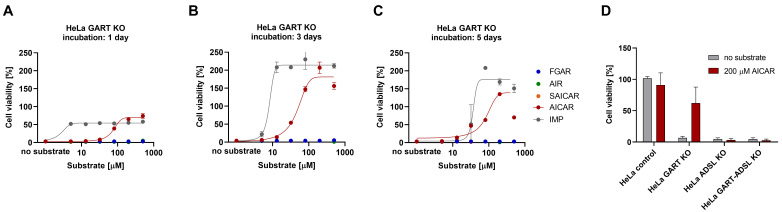
AICAR restores the viability of HeLa GART KO cells. The viability of HeLa GART KO cells was measured after (**A**) 1 day of incubation, (**B**) 3 days of incubation, (**C**) 5 days of incubation in purine-depleted medium with FGAR (blue), AIR (green), SAICAR (orange), AICAR (red), IMP (grey) in concentration range from 5 μmol/L to 500 μmol/L. (**D**) HeLa control, GART KO, ADSL KO, GART-ADSL KO viability measurement after 72 h incubation in normal medium containing no metabolite (grey) or 200 μM AICAR (red). Each data point represents the mean of 3 measurements. Vertical bars represent S.D. (n = 3).

The interesting finding that AICAR did not reduce the viability of tested cell lines led us to further investigate this phenomenon. We used HeLa GART KO cells deficient in trifunctional enzyme glycineamide ribonucleotide synthetase/aminoimidazole ribonucleotide synthetase/glycineamide ribonucleotide transformylase (GART), ADSL KO cells deficient in bifunctional ADSL enzyme, and GART-ADSL double knockout cells (GART-ADSL KO). These KO cells survive only with purines added to growth medium [42]. First, we tested HeLa GART KO cells in purine-depleted (PD) medium supplemented with FGAR, AIR, SAICAR, AICAR, and IMP for one, three, or five days (Figure 4A–C) and monitored whether the cell viability was restored. IMP served as a positive control that restores the viability of GART KO cells.

FGAR did not preserve the viability of GART KO cells, which supported the results of the cell viability experiment, where it reduced the cell viability of tested cell lines. Although AIR entered the cells and can probably enter the purinosome, we did not observe any phenotype rescue, which, along with cell viability experiments, suggests its cytotoxic effect on cellular metabolism. SAICAR also did not rescue the phenotype of GART KO cells, which is in line with the results of the flux experiment. Only AICAR, which we detected to be nontoxic and able to enter the cells, restored the viability of GART KO cells similar to IMP. We conducted the experiment in HeLa GART KO, ADSL KO, and GART-ADSL KO that we treated by 200 µM AICAR under normal growth conditions for three days. We found that viability was not restored in HeLa ADSL KO and GART–ADSL KO cells due to the interrupted second catalytic function of ADSL in the PNC that produces AMP under physiological conditions. We detected increased viability of HeLa GART KO (Figure 4D), indicating that added AICAR increases viability of the cells through restoration of adenylate pool.

## 4. Discussion

In this investigation, we tested the uptake and flux of the isotopically labeled DNPS metabolites FGAR, AIR, SAICAR, and AICAR in HeLa cells. We found that FGAR and SAICAR are not processed or only poorly processed through DNPS, while AIR and AICAR are metabolized into products of DNPS, as well as recycling and degradation pathways. In the case of patients, the accumulation of DNPS metabolites is considered toxic and results in clinical sequelae [6]. Therefore, we tested the cell viability in the presence of FGAR, AIR, SAICAR, and AICAR and their dephosphorylated counterparts, FGAr, AIr, SAICAr, and AICAr, in different cell types. Patients with ADSL deficiency show reduced enzyme activity in isolated tissues and massive accumulation of SAdo and SAICAr in urine, blood, and CSF [5,8,13,14]; patients with AICAribosiduria show accumulation of AICAr, SAdo, and SAICAr in body fluids [2,4]. The expected significant toxic effect of these metabolites was not confirmed. The metabolic profile of ADSL deficiency differs greatly from the mild form (SAICAr: 125–390 µM, SAdo: 260–1130 µM), severe form (SAICAr: 111–953 µM, SAdo: 120–649 µM) to the neonatal form (SAICAr: 164–1100 µM, SAdo: 160–684 µM) in CSF [11]; thus, the absolute concentrations of SAICAr and SAdo were insufficient to establish the ADSL deficiency phenotype. For this reason, it has been proposed that the SAdo/SAICAr ratio, measured in the CSF, correlates with the patient’s development. Mild forms are characterized by a ratio > 2, severe forms by a ratio ~1, and neonatal forms by a ratio < 1 [5,23,49]. With these observations came the question of whether SAdo is the protective intermediate in ADSL deficiency and SAICAr is the harmful metabolite. Our results revealed a slightly elevated cytotoxic effect of SAICAR treatment in all tested cell lines and a stimulatory response in CAD 5 and K562. SAICAr exhibited the most cytotoxic effect in HeLa cells (IC_50_ = 424 µM), and we also elicited a stimulatory response in CAD 5 (<14.3 µM) [44,50] and K562 cells (<24.4 µM). Based on our flux analysis in HeLa control and ATIC KO cells, where we detected SAICAr*, AICAR*, and AICAr*, we postulate that SAICAR is first dephosphorylated at the outer cellular membrane to SAICAr*, and the latter is transferred into the cytoplasm by a concentrative nucleoside transporter (CNT) or equilibrative nucleoside transporter (ENT). Our assumption is consistent with studies of different ectonucleotidases, CNTs, and ENTs [44,50].

SAICAR can also form a complex with PKM2 and activate pyruvate kinase activity of PKM2 in cancer cells under glucose deprivation [28,51]. Depletion of ADSL in hepatocellular carcinoma upregulates activating transcription factor 4 (ATF4) and induces mitochondrial stress; however, the effect of SAICAR has not been examined [37].

Because the clinical manifestations of ADSL deficiency are unrelenting, we think that there may exist another mechanism of toxicity besides the accumulation of DNPS metabolites. Previously, it was shown that the total nucleotide pool concentrations are not reduced in patients [6,8,14,15], and the ATP concentration was 6.4 nmol/10^6^ cells in control skin fibroblasts and 7.3 nmol/10^6^ cells in fibroblasts of severe ADSL deficiency [15]. In line with these findings are our unpublished measurements where we did not detect any decrease in nucleotide pools in ADSL deficient fibroblasts. Therefore, the pathogenesis of ADSL deficiency does not appear related to the impaired total nucleotide pool concentrations but rather by metabolic imbalance.

It has been postulated that adenosine acts as crucial regulator of the neurotransmitter system in the brain and that alterations in purine catabolism and anabolism lead to pathologic neurological outcomes [52,53]. The purinergic receptors are heterogeneously expressed in many tissues in the body, and the receptors that primarily bind adenosine are expressed in nervous tissue and brain [54,55]. The transport of various ribosides to the choroid plexus, where CSF is produced, is facilitated by ENTs and CNTs [56,57]. We postulate that metabolic imbalance more likely contributes to toxicity through similarity with adenosine and possible competition of DNPS metabolites for the same purinergic receptor.

Patients with AICAribosiduria develop urinary AICAr concentrations of 212 mmol/mol of creatinine and SAICAr, SAdo of approximately 50 mmol/mol of creatinine. In red blood cells were detected AICAR (243 mmol/mL) and its phosphorylated counterparts: ZDP (AICAdiphosphate, 224 mmol/mL) and ZTP (AICAtriphosphate, 716 mmol/mL) [2,4]. Marie et al. demonstrated a lower concentration of ATP, concurrently with higher concentrations of AMP and GMP in red blood cells from the patient with AICAribosiduria [2].

Although Douillet et al. [58] showed that AICAR (ZMP) is probably toxic to yeast cell growth, our study shows an inhibitory response of AICAR and AICAr only at a concentration greater than 600 µmol/L. Additionally, at lower concentrations <600 µmol/L of AICAR and AICAr, we observed increased viability in all cell lines, especially in HepG2 and K562 cells. Our findings are supported by Scudiero et al. [59], who described the low cytotoxic effects of AICAR and AICAr (in the publication described as AICAR) in human cell lines. Treatment of several cell lines (NIH/3T3, J774, OMK, HepG2/C3A, hepatocytes) with 1 mM AICAr has shown improved cryopreservation and accumulation of different amounts of AICAR, AICAr, ZDP, and ZTP [60]. Our data demonstrated that with an increasing concentration (0–500 μM) of AICAR, GART KO cells restored viability in purine-depleted medium, and that under normal growth conditions, they improved viability in the presence of 200 μM AICAR. However, ADSL KO and GART-ADSL KO were unable to rescue their phenotype due to the dual function of the ADSL enzyme in the DNPS and in PNC, where it converts SAMP into AMP. We believe that AICAR contributes to the rescue of GART KO by entering DNPS and establishing the AMP and GMP pools. Furthermore, elevated AICAR concentration leads to allosteric activation of AMP-activated protein kinase (AMPK) that regulates the intracellular AMP:ATP ratio [25,26,27,30,32]. We confirmed that AICAR* enters the cell after its dephosphorylation to AICAr* and is most likely transported by the equilibrative adenosine transporter, as shown by Gadalla et al. [61]. Once AICAr* enters the cell, it is phosphorylated to AICAR*, presumably by adenosine kinase [62], and further metabolized into AICAr*, IMP*, inosine*, xanthine*, and SAdo*. Interestingly, we were able to detect the upstream metabolite SAICAR* and SAICAr* in cell lysate of ATIC KO cells and SAICAr* in growth medium of ATIC KO cells. These findings indicate that AICAR* enters the cell and is processed by two DNPS reactions, namely the forward reaction by the ATIC enzyme and the backward reaction by the ADSL enzyme. The ADSL reverse reaction is highly activated in ATIC KO cells that naturally accumulate AICAR, and most probably also in the cells of AICAribosiduria patients. Taken together, we hypothesize that the pathogenicity of AICAribosiduria is not only the result of the accumulation of AICAR or AICAr.

ATIC overexpression in hepatocellular carcinoma cells enhances their proliferation. On the other hand, downregulation of ATIC results in autophagy and apoptosis [40]. Knowing that AICAR has an impact on the majority of cellular processes [59] and SAICAR is a partner of the PKM2 enzyme [28,61], we can speculate that toxicity is due to an entire cascade of metabolic changes rather than to the accumulation of metabolites themselves [28,51,61].

Little is known about AIR. In bacteria, AIR is a precursor of thiamine, but this reaction is not confirmed in higher organisms [63]. The newly identified disorder, PAICS deficiency, is associated with low PAICS activity and no accumulation of AIr in patient skin fibroblasts [3]. A high level of AIr was found in HeLa PAICS KO cell medium [42], and the cytotoxic effect of AIr was detected in CAD 5 cells and control skin fibroblasts [3]. Our data show that AIR is processed by DNPS. We found AIr* in HeLa cell media, demonstrating AIR* dephosphorylation at the outer cellular membrane. AIr* is transported via a nucleoside transporter into the cytoplasm of the cell and metabolized into AICAr*, which was found in medium and inosine*, which was found in cell lysate. ATIC KO cells metabolize AIR* into final metabolites AICAR* and AICAr*. Even though AICAR* appears in the cell lysate of ATIC KO cells and AICAr* appears in HeLa control media, AIR treatment did not enhance the survival of GART KO cell under purine-depleted conditions. The cytotoxicity study revealed IC_50_ values lower than those of SAICAR and SAICAr. Similar viability curves were found for AIR and AIr in all cell lines and in CAD 5, K562, and in skin fibroblasts, an unexpected increase of viability was demonstrated. Therefore, we hypothesize that due to its small molecular weight, it may act at different concentrations as a molecular modulator or inhibitor. Furthermore, PAICS knockdown (KD) in breast cancer cell lines and lung adenocarcinoma cells resulted in reduced cell viability and proliferation, and cell cycle disruption [34,39]. The expression of proteins involved in cell cycle regulation and apoptosis differs in PAICS KD breast cancer cells [39]. However, the contribution of PAICS reaction substrates and products to cell viability and the expression profile of other interaction partners has not been studied. Since AIR contains an imidazole moiety that has been investigated for its anticancer effect, our findings may contribute to elucidate the inhibition of cellular metabolism [64].

Recently, using quantitative measurement of purine intermediates in urine, we identified a patient with an elevated concentration of FGAr, indicative of PFAS deficiency [1], and elevated concentration of FGAr in growth medium of HeLa PFAS KO cells [42]. Based on our flux experiment, FGAR* likely dephosphorylates at the outer cellular membrane into FGAr* and is transferred via nucleoside transporter into the cell. We hypothesize that FGAR* cannot enter the active site of the PFAS as a result of close proximity of amidophosphoribosyl transferase (PPAT), GART, and PFAS enzymes, the core of the purinosome [19]. This theory is supported by measurements of the flux of DNPS in HeLa cell lysate treated with ^13^C-glycineamide ribotide (GAR*) where we detect only FGAR* (unpublished results). Our cytotoxicity study revealed that FGAR inhibited viability in different cell types and that FGAr and FGAR in PD medium did not rescue the phenotype of GART KO cells. The toxic effect must be the result of a complex metabolic response.

In summary, we found that AIR, SAICAR, and AICAR are partially or fully processed by DNPS, and although our cytotoxicity data do not correspond to the finding of concentrations of DNPS metabolites in body fluids, we propose an alternative mechanism of toxicity. We postulate that the dephosphorylated metabolite is exported by the affected cell and accumulates in the extracellular space, where it binds to the receptor of susceptible cells, leading to a cascade of reactions resulting in metabolic imbalance and eventually cell death. The study of ENTs and CNTs and other transporters would be beneficial for monitoring transport of DNPS metabolites and receptor–ligand binding assays to determine interaction partners for DNPS metabolites.

## 5. Conclusions

In conclusion, we provide new information about DNPS metabolites, which accumulate massively in body fluids under pathological conditions and are considered neurotoxic. For the first time, we studied the cell viability in the presence of FGAR and AIR. We examined the fate of FGAR, AIR, SAICAR, and AICAR within DNPS, the salvage pathway, and PNC. We proved that AIR, SAICAR, and AICAR enter HeLa control and ATIC KO cells. Here we propose that DNPS intermediates act as metabolic modulators, and their toxic effect is much more complex than direct toxicity of the accumulated metabolites. Further studies of receptor–ligand interaction and nucleoside/nucleotide transporters can lead to novel insights into pathogenesis and therapy.

## Figures and Tables

**Table 1:** IC_50_ values of DNPS metabolites.

	IC_50_ (μM)				
Metabolite/Cells	HeLa	CAD 5	HepG2	K562	Fibroblasts
**FGAR**	396	292	816	270	385
**FGAr**	270	181	186	181	323
**AIR**	219	313	264	341	466
**AIr**	157	332 [3]	237	337	120 [3]
**SAICAR**	1532	1269	1396	1506	2957
**SAICAr**	424	618 [3]	509	823	1804 [3]
**AICAR**	1726	2132	– ^†^	– ^†^	1554
**AICAr**	785	1146	– ^†^	902	1478

^†^ Only stimulatory response was detected.

## Data Availability

The data that support the findings of this study are available in the methods and/or Supplementary Materials of this article. “Unpublished results” are available upon request from the corresponding or first authors.

## Supplementary Materials

The following supporting information can be downloaded at: https://www.mdpi.com/article/10.3390/metabo12121210/s1, Figure S1: Proposed model of distribution of isotopically labeled DNPS metabolites (FGAR*, AIR*, SAICAR*, AICAR*) within HeLa control and HeLa ATIC KO cells; Figure S2: FGAR* and FGAr* treatment of HeLa control and ATIC KO cells; Figure S3: Representative chromatograms of isotopically labeled DNPS metabolites in HeLa control and HeLa ATIC KO cell lysate, media, and blank medium after treatment with AIR*; Table S1: Selected reaction monitoring (SRM) of DNPS metabolites.
