# Supplementary material for: Metabolites of De Novo Purine Synthesis: Metabolic Regulators and Cytotoxic Compounds
Source: Metabolites. 2022 Dec 2;12(12):1210. doi: 10.3390/metabo12121210 (PMC9788633; doi:10.3390/metabo12121210)
Supplement: Supplementary file 1 [file metabolites-12-01210-s001.zip › metabolites-1991338-supplementary/Figures.pdf]

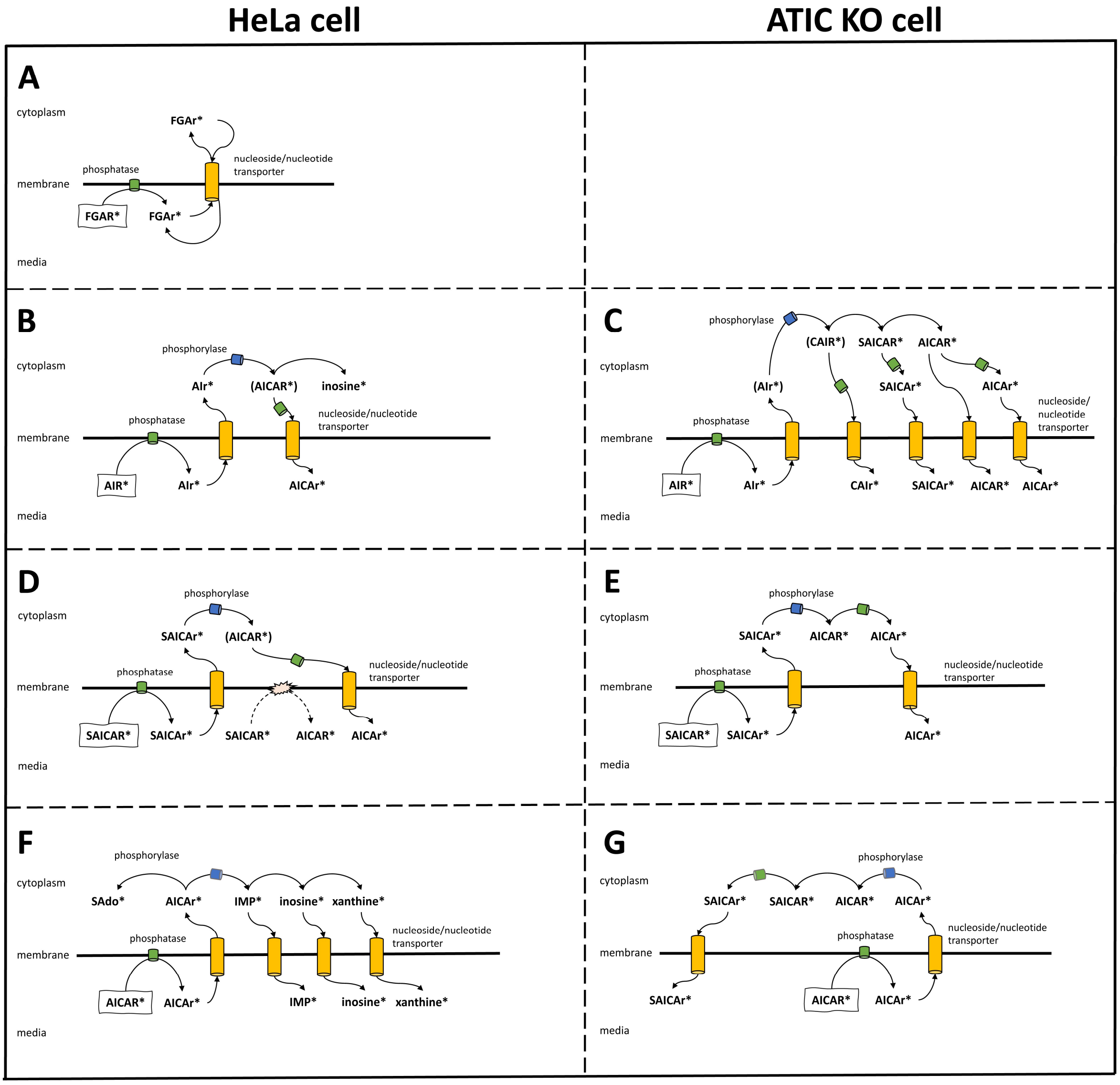

Figure S1. Proposed model of distribution of isotopically labelled DNPS metabolites (FGAR*, AIR*, SAICAR*, AICAR*) within HeLa control and HeLa ATIC KO cells. (A) Uptake of FGAR* from the medium into the cytoplasm of HeLa results in dephosphorylation to FGAr*. (B) The uptake of AIR* from the media into the cytoplasm of HeLa cells results in the synthesis of AIr*, SAICAR*, SAICAr*, and inosine*. (C) Uptake of AIR* from the media into the cytoplasm of HeLa ATIC KO cells results in the synthesis of AIr*, CAIR*, CAIr*, SAICAR*, SAICAr*, AICAR* and AICAr*. (D) Uptake of SAICAR* from the medium into the cytoplasm of HeLa cells results in the synthesis of SAICAr*, AICAR*, and AICAr*. The transformation from SAICAR* to AICAR* in extracellular space is shown with a dashed arrow. (E) The uptake of SAICAR* from the media into the cytoplasm of ATIC KO cells results in the synthesis of SAICAr*, AICAR*, and AICAr*. (F) Uptake of AICAR* from the media into the cytoplasm of HeLa cells results in the synthesis of AICAr*, IMP*, inosine*, xanthine*, and SAICAr*. (G) The uptake of AICAR* from the media into the cytoplasm of ATIC KO cells results in the synthesis of AICAr*, SAICAR*, and SAICAr*. Isotopically labelled metabolites are displayed with asterisks. The metabolites in the brackets were not detected, but we assume their presence during metabolite processing. The nucleoside/nucleotide transporter is shown as a long yellow tube. Phosphatase is shown as a short green tube. Phosphorylase is shown as a short blue tube. Administrated metabolite has a black border.

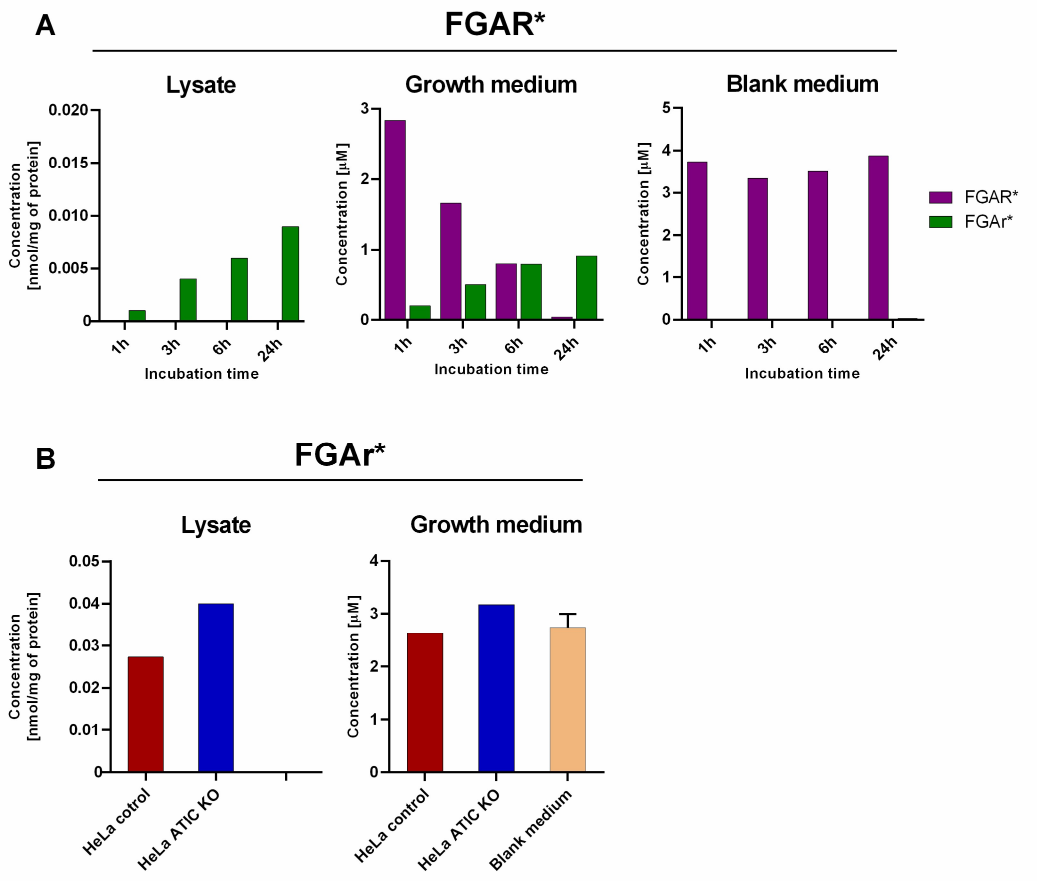

Figure S2. FGAR* and FGAr* treatment of HeLa control and ATIC KO cells. The graphs (A) represent treatment of HeLa cells with FGAR* in different timepoints. Individual graphs show detected metabolites FGAR* (purple) and FGAr* (green) in cell lysate and medium. The graphs (B) show detection of FGAr* in cell lysate and medium of HeLa control cells (red) and ATIC KO cells (blue) after treatment with FGAr* for 6 hours. Orange column shows blank medium with FGAr*.

A) HeLa cells

AIR*

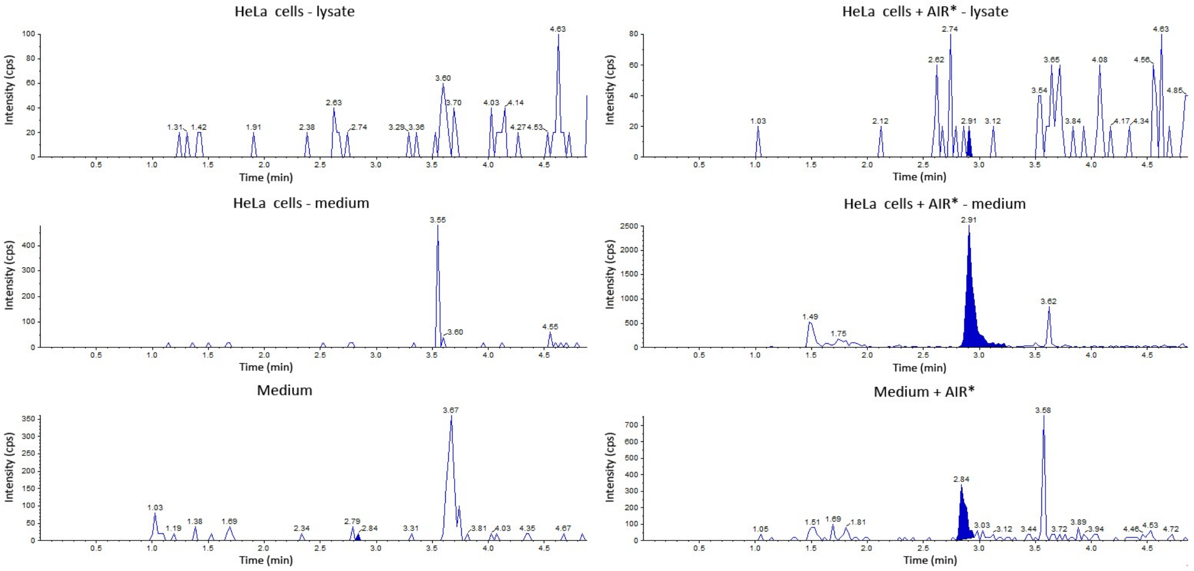

AIr*

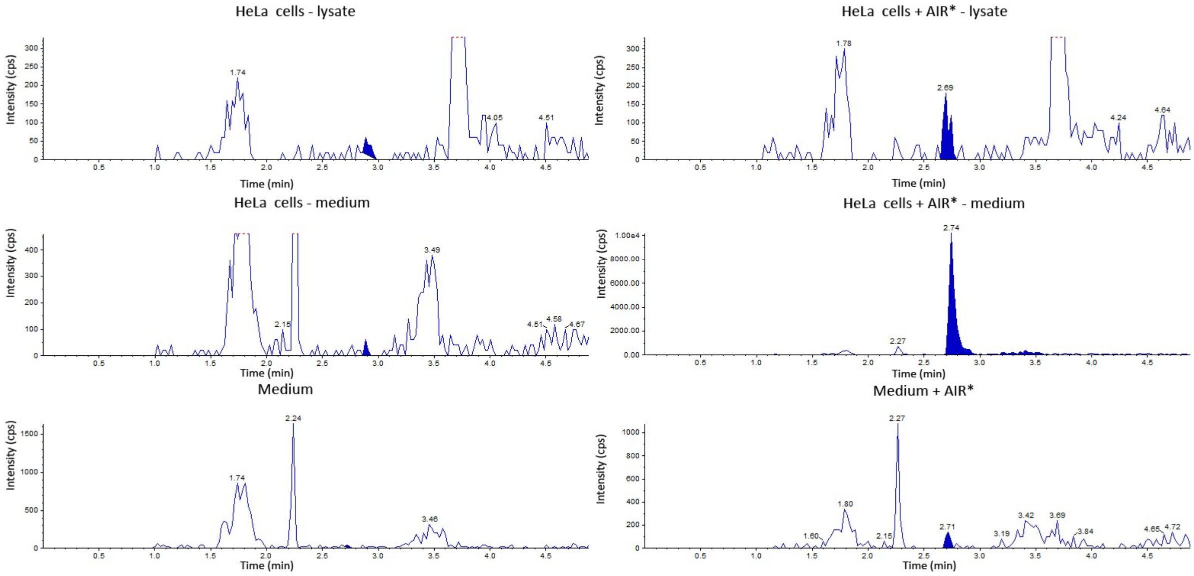

AICAr*

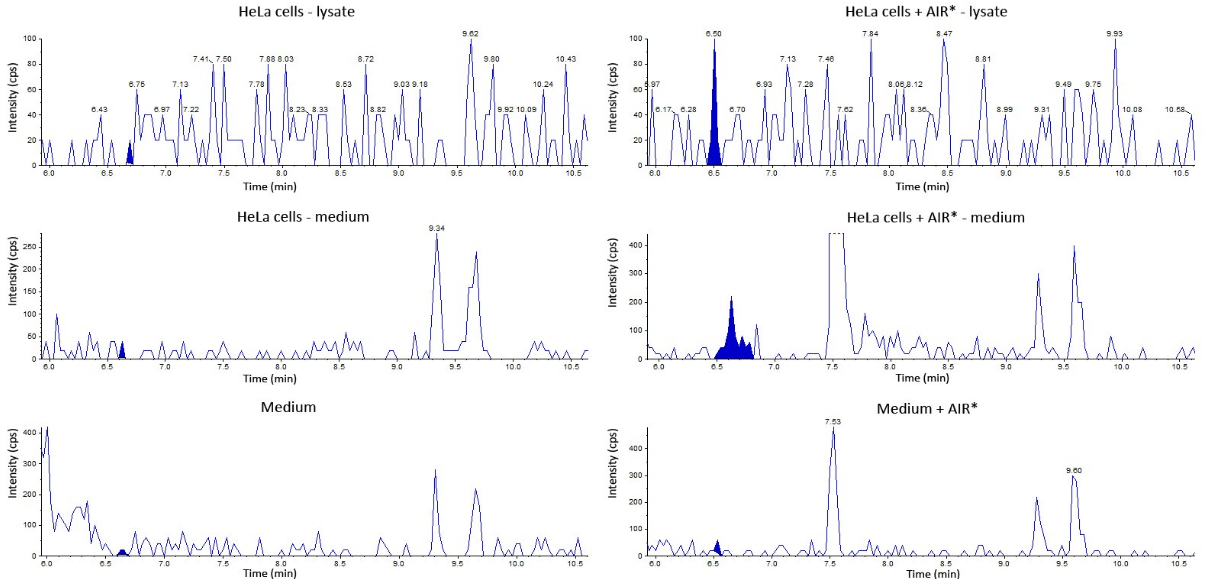

inosine*

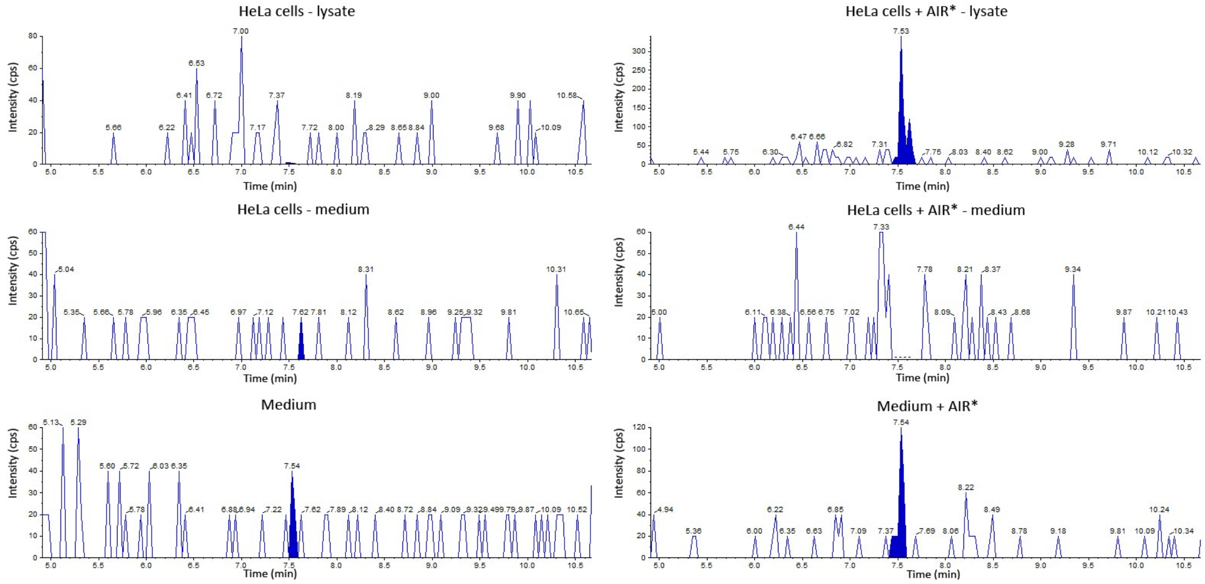

B) HeLa ATIC KO

AIR*

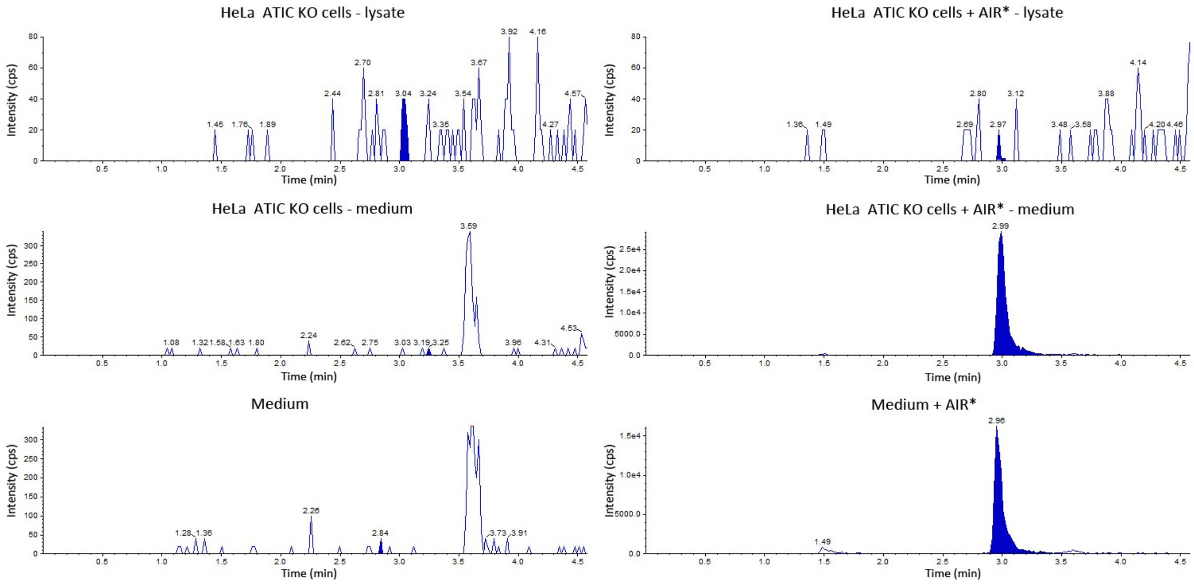

AIr*

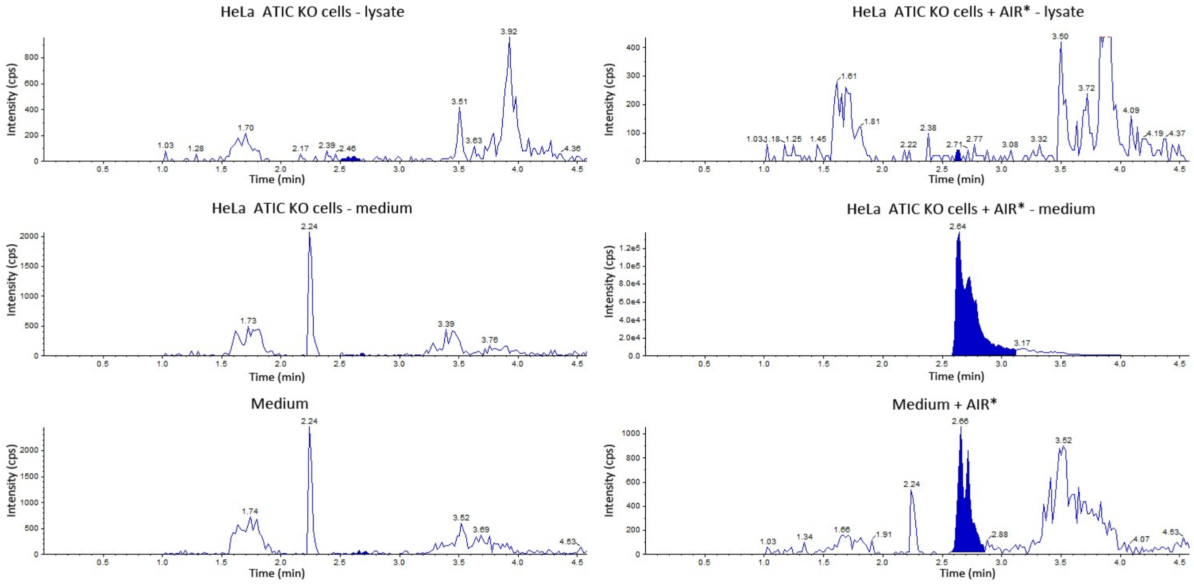

CAIr*

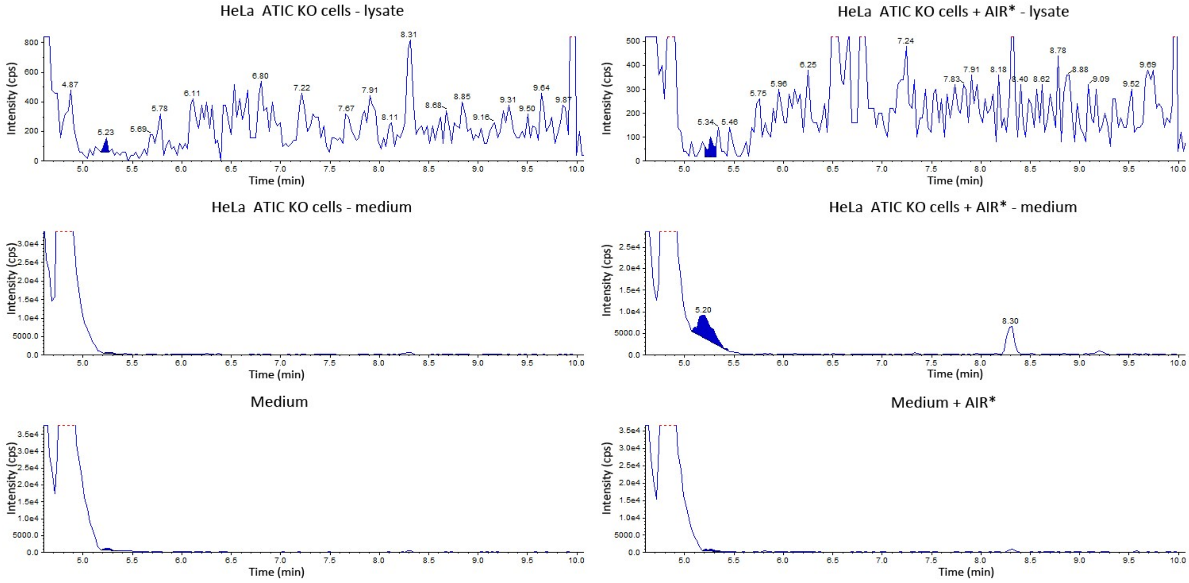
HeLa ATIC KO cells - lysate
HeLa ATIC KO cells + AIR* - lysate
HeLa ATIC KO cells - medium
HeLa ATIC KO cells + AIR* - medium
Medium
Medium + AIR*
Intensity (cps)
Time (min)

SAICAR*

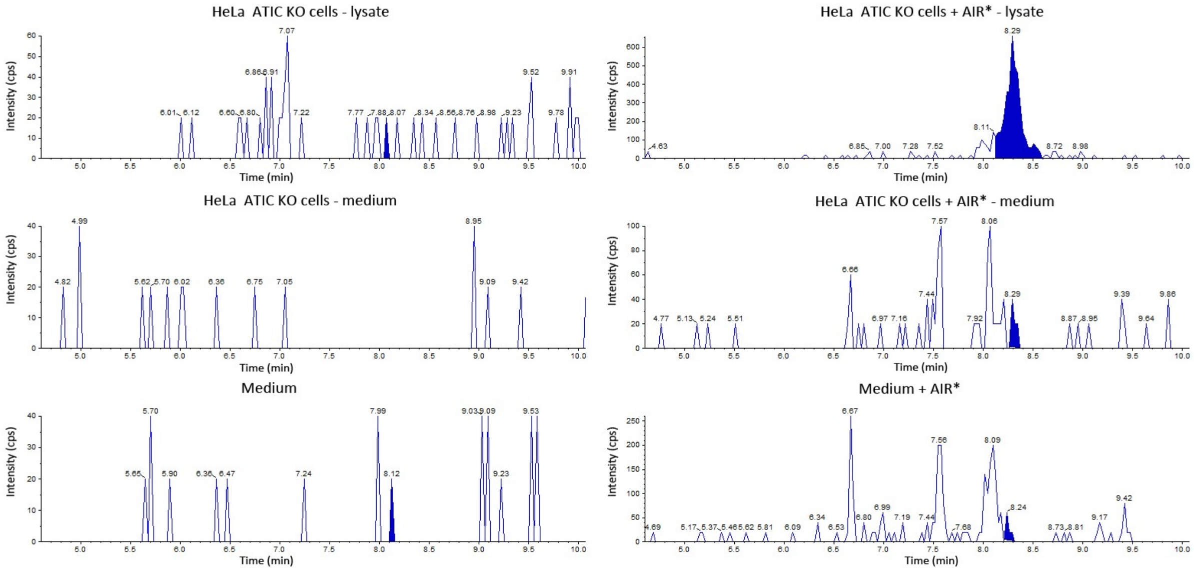
HeLa ATIC KO cells - lysate
HeLa ATIC KO cells + AIR* - lysate
HeLa ATIC KO cells - medium
HeLa ATIC KO cells + AIR* - medium
Medium
Medium + AIR*
Intensity (cps)
Time (min)

SAICAr*

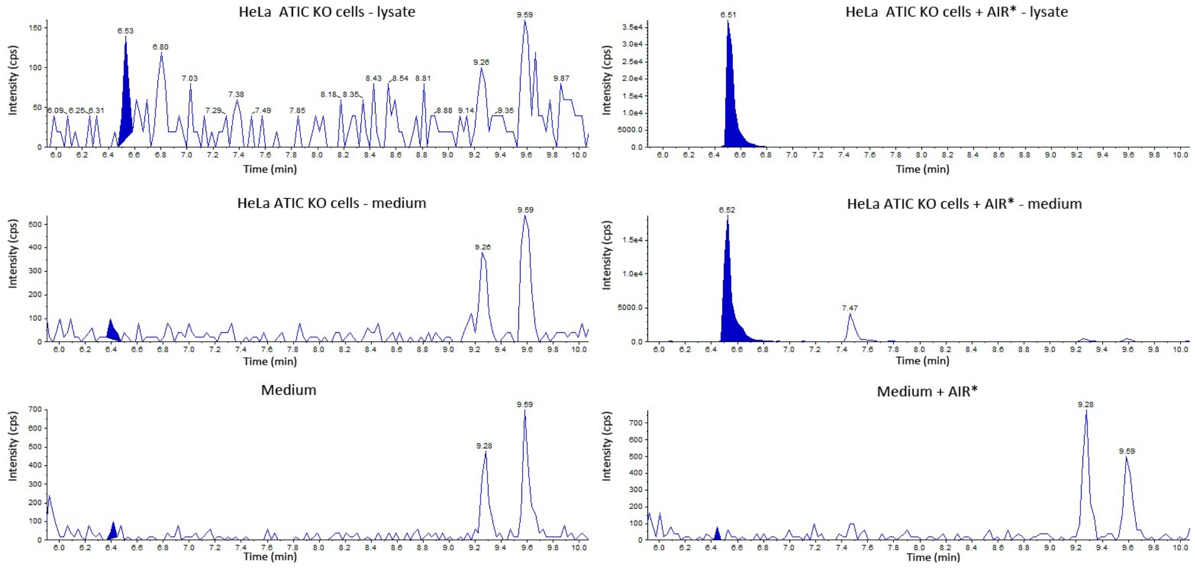

AICAR*

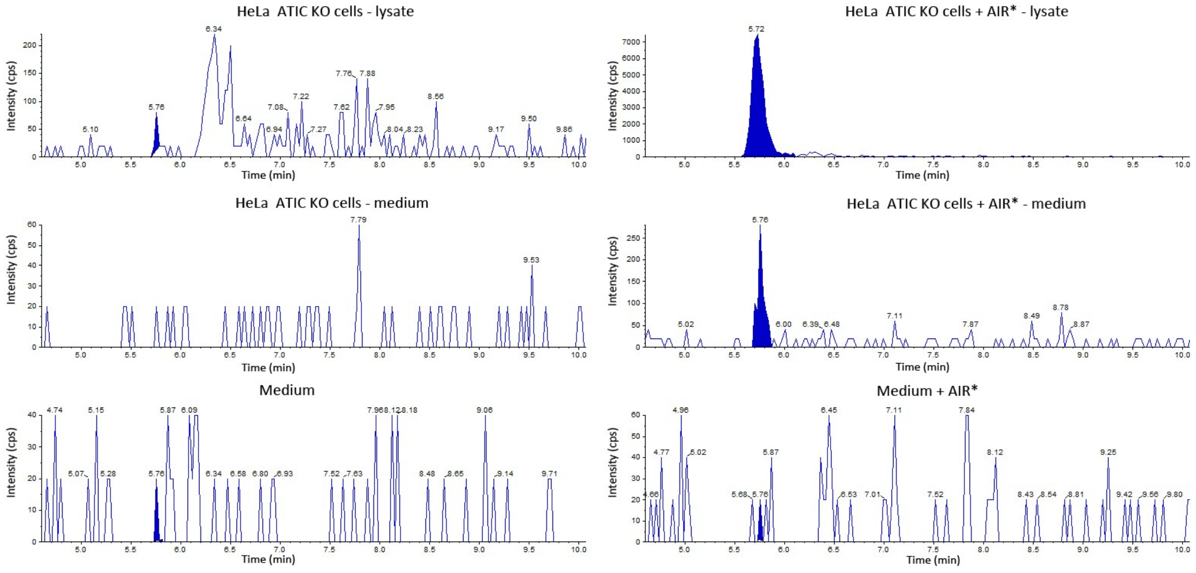

AICAr*

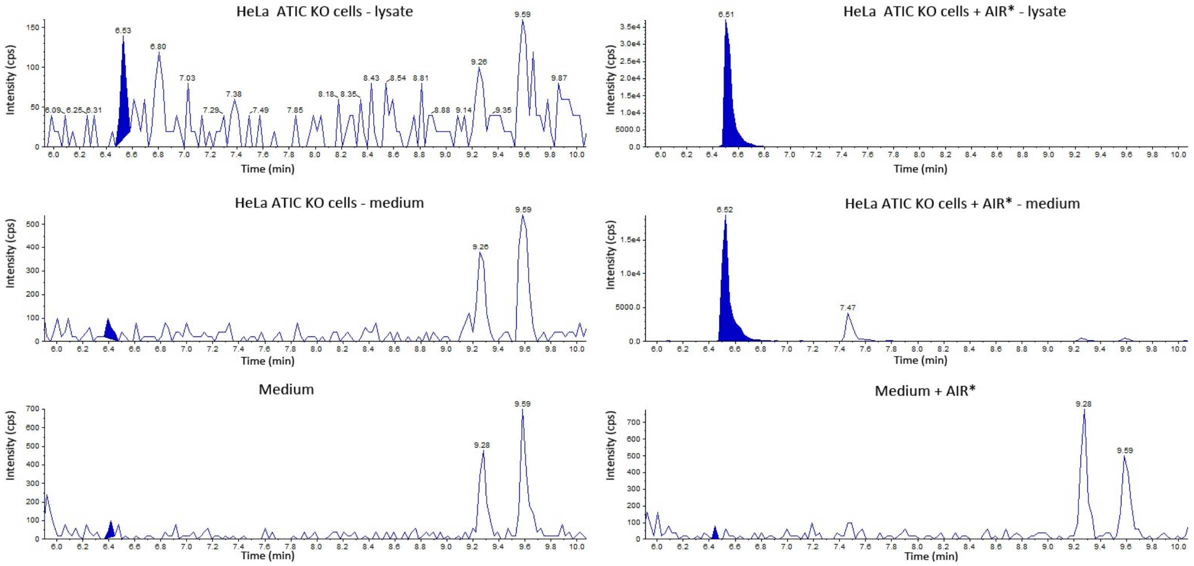

Figure S3. Representative chromatograms of isotopically labelled DNPS metabolites in HeLa control and HeLa ATIC KO cell lysate, media, and blank media after treatment with AIR*. Chromatograms show identified isotopically labelled purine metabolites (marked with *) in A) HeLa control and B) HeLa ATIC KO cell lysate, the growth media and blank media. The header of each set of chromatograms represents quantified isotopically labelled purine metabolite. Quantified metabolites are shown in solid blue color. Left column carries information for untreated cells or blank media and right column carries information for treated cells with AIR* or blank media treated with AIR*. Axis-x shows Time (min), and axis-y shows signal intensity expressed as counts per second (CPS).
