# Supplementary material for: Metabolites of De Novo Purine Synthesis: Metabolic Regulators and Cytotoxic Compounds
Source: Metabolites. 2022 Dec 2;12(12):1210. doi: 10.3390/metabo12121210 (PMC9788633; doi:10.3390/metabo12121210)
Supplement: Supplementary file 1 [file metabolites-12-01210-s001.zip › metabolites-1991338-supplementary/Table_S1.pdf]

Table S1: Selected reaction monitoring (SRM) of DNPS metabolites

STANDARD			Treatment with $^{13}C_2$ - FGAR			Treatment with $^{13}C_2$,^{15}N - AIR			Treatment with $^{13}C_2$,$^{15}N_2$ - SAICAR			Treatment with $^{13}C_2$,$^{15}N_2$ - AICAR		
name	MS1	MS2	name	MS1	MS2	name	MS1	MS2	name	MS1	MS2	name	MS1	MS2
**GAR**	287.1	75.1	**$^{13}C_2$-GAR**	289.1	77.1	**$^{13}C_2$-GAR**	289.1	77.1						
**GAr**	207.1	75.1	**$^{13}C_2$-GAr**	209.1	77.1	**$^{13}C_2$-GAr**	209.1	77.1						
**FGAR**	315.1	103	**$^{13}C_2$-FGAR**	317.1	105	**$^{13}C_2$-FGAR**	317.1	105						
**FGAr**	235.1	103.1	**$^{13}C_2$-FGAr**	237.1	105.1	**$^{13}C_2$-FGAr**	237.1	105.1						
**FGAMR**	314.2	102,1	**$^{13}C_2$-FGAMR**	316.2	104.1									
**FGAMr**	234.1	102.1	**$^{13}C_2$-FGAMr**	236.1	104.1									
**AIR**	296.2	84	**$^{13}C_2$-AIR**	298.2	86	**$^{13}C_2$,^{15}N-AIR**	299.2	87						
**AIr**	216.2	84	**$^{13}C_2$-AIr**	218.2	86	**$^{13}C_2$,^{15}N-AIr**	219.2	87						
**CAIR**	340.2	128.2	**$^{13}C_2$-CAIR**	342.2	130.2	**$^{13}C_2$,^{15}N-CAIR**	343.1	131.2						
	340.2	110.2				**$^{13}C_2$-CAIR**	342.1	130.2						
**CAIr**	260.2	128.2	**$^{13}C_2$-CAIr**	262.2	130.2	**$^{13}C_2$,^{15}N-CAIr**	263.1	131.2						
**SAICAR**	455.2	243.0	**$^{13}C_2$-SAICAR**	457.2	245.0	**$^{13}C_2$,^{15}N-SAICAR**	458.2	246.0	**$^{13}C_2$,$^{15}N_2$-SAICAR**	459.2	247.0	**$^{13}C_2$,$^{15}N_2$-SAICAR**	459.2	247.0
**SAICAr**	375.2	243.0	**$^{13}C_2$-SAICAr**	377.2	245.0	**$^{13}C_2$,^{15}N-SAICAr**	378.2	246.0	**$^{13}C_2$,$^{15}N_2$-SAICAr**	379.2	247.0	**$^{13}C_2$,$^{15}N_2$-SAICAr**	379.2	247.0
**AICAR**	339.2	110.2	**$^{13}C_2$-AICAR**	341.2	112.2	**$^{13}C_2$,^{15}N-AICAR**	342.2	113.2	**$^{13}C_2$,$^{15}N_2$-AICAR**	343.2	113.2	**$^{13}C_2$,$^{15}N_2$-AICAR**	343.2	113.2
	339.2	127.2		341.2	129.2					343.2	131.2		343.2	131.2
**AICAr**	259.2	127.2	**$^{13}C_2$-AICAr**	261.2	129.2	**$^{13}C_2$,^{15}N-AICAr**	262.2	130.2	**$^{13}C_2$,$^{15}N_2$-AICAr**	263.2	131.2	**$^{13}C_2$,$^{15}N_2$-AICAr**	263.2	131.2
**FAICAR**	367.1	155.1	**$^{13}C_2$-FAICAR**	369.1	157.1	**$^{13}C_2$,^{15}N-FAICAR**	370.1	158.1				**$^{13}C_2$,$^{15}N_2$-FAICAR**	371.1	159.2
**FAICAr**	287.2	138.2	**$^{13}C_2$-FAICAr**	289.2	140.2	**$^{13}C_2$,^{15}N-FAICAr**	290.2	141.2				**$^{13}C_2$,$^{15}N_2$-FAICAr**	291.2	141.1
													291.2	142.1
**IMP**	349.1	137.0	**$^{13}C_2$-IMP**	351.1	139	**$^{13}C_2$,^{15}N-IMP**	352.1	140	**$^{13}C_2$,$^{15}N_2$-IMP**	353.1	141	**$^{13}C_2$,$^{15}N_2$-IMP**	353.1	141
**inosine**	269.0	137.0	**$^{13}C_2$-inosine**	271.0	139.0	**$^{13}C_2$,^{15}N-inosine**	272.0	140.0	**$^{13}C_2$,$^{15}N_2$-inosine**	273.0	141.0	**$^{13}C_2$,$^{15}N_2$-inosine**	273.0	141.0
**xanthine**	153.1	110.1	**$^{13}C_2$-xanthine**	155.1	112.1	**$^{13}C_2$,^{15}N-xanthine**	156.1	113.1	**$^{13}C_2$,$^{15}N_2$-xanthine**	157.1	139.0	**$^{13}C_2$,$^{15}N_2$-xanthine**	157.1	139.0
	153.1	136.0		155.1	138								157.1	113.1
**hypoxanthine**	137.1	110.1	$^{13}C_2$-hypoxanthine	139.1	112.1	$^{13}C_2$,^{15}N-hypoxanthine	140.1	113.1	$^{13}C_2$,$^{15}N_2$-hypoxanthine	141.1	114.1	$^{13}C_2$,$^{15}N_2$-hypoxanthine	141.1	114.1
	137.1	119.0		139.1	121.0					141.1	123		141.1	123
										141.1	113.1		141.1	113.1
**SAdo**	384.2	252	**$^{13}C_2$-SAdo**	386.2	254	**$^{13}C_2$,^{15}N-SAdo**	387.2	255.1				**$^{13}C_2$,$^{15}N_2$-SAdo**	388.2	256.1
**SAMP**	464.1	251.7	**$^{13}C_2$-SAMP**	466.1	253.7	**$^{13}C_2$,^{15}N-SAMP**	467.1	254.7				**$^{13}C_2$,$^{15}N_2$-SAMP**	468.1	255.7
**uric acid**	169.3	141.0	**$^{13}C_2$-uric acid**	171.3	143.0	**$^{13}C_2$,^{15}N-uric acid**	172.3	144.0	**$^{13}C_2$,$^{15}N_2$-uric acid**	173.3	145.0	**$^{13}C_2$,$^{15}N_2$-uric acid**	173.3	145.0
